# A prospective study of pre-trauma fear learning and extinction as risk factors for posttraumatic stress disorder

**DOI:** 10.1016/j.xjmad.2025.100148

**Published:** 2025-09-13

**Authors:** Dean T. Acheson, Jonathon R. Howlett, Katia M. Harlé, Daniel M. Stout, Dewleen G. Baker, Caroline M. Nievergelt, Mark A. Geyer, Victoria B. Risbrough, MRS-II Team

**Affiliations:** a Department of Psychiatry, University of California San Diego, 9500 Gilman Dr. Mail Code 0804, La Jolla, CA 92093-0804, United States; b Center for Excellence in Stress and Mental Health, United States; c Mental Illness Research, Education and Clinical Center, VA San Diego Healthcare System, 3350 La Jolla Village Drive, San Diego, CA 92161, United States

**Keywords:** PTSD, Fear Extinction, Fear Conditioning, Longitudinal

## Abstract

Identifying risk for developing trauma-related disorders is a critical step in future prevention and intervention strategies. Impairments in inhibition of learned fear, such as safety signal learning and fear extinction, are suggested to be core mechanisms underlying symptom development and maintenance of posttraumatic stress disorder (PTSD). However, it is unclear if these impairments are pre-existing risk factors for PTSD, or if fear inhibition abnormalities arise only after trauma and symptom development. We utilized a prospective-longitudinal study in human, male service members at high risk for trauma exposure to test the hypothesis that learned fear impairments are pre-existing risk factors for PTSD. PTSD symptoms, fear learning, and fear extinction were assessed prior to a 7-month combat deployment to Afghanistan and 4–6 months after return (final N = 643). Fear learning and extinction were measured by fear-potentiated startle, self-reported anxiety, and threat expectancy ratings. Poor discrimination between threat and safety signals before trauma predicted higher likelihood for development of new onset PTSD after trauma, but not PTSD severity. This effect remained when controlling for trauma exposure. Lower fear extinction learning rate at the pre-trauma time-point predicted PTSD severity but not PTSD status. These findings support the hypothesis that both overgeneralization of fear and/or poor safety signal learning as well as slower fear extinction learning may predispose individuals for development of PTSD. These findings support further study of cue discrimination and slow fear extinction learning as “intermediate phenotypes” for intervention strategies and mechanistic studies targeting the neurobiology of risk and resilience to trauma-related disorders.

## Highlights

• Cue discrimination/generalization predicts development of PTSD after trauma exposure.
• Slower fear extinction learning is associated with PTSD severity after trauma.
• Cue discrimination and slow extinction learning may play a mechanistic role in trauma processing.

## 1 Introduction

The mechanisms underlying the etiology and maintenance of trauma-related disorders are largely unknown. However, there is consensus that many independent mechanisms may underlie these conditions, leading to heterogeneity of symptom development and expression [1]. Linking mechanistic disruptions to symptom patterns is a crucial step in developing targeted prevention and treatment efforts, as well as predicting treatment response [2], [3]. One potential mechanism potentially contributing to risk for and maintenance of trauma-related symptoms is differences in trait fear learning and inhibition [4], [5], [6].

The “Marine Resiliency Study II” (MRS-II; Oct 2011-Oct 2013) is a longitudinal investigation of neurocognitive performance in service members (Marines and Navy personnel) deployed to Afghanistan, with the purpose of identifying markers of risk/resiliency for trauma-related disorders. Individuals with PTSD are consistently shown to exhibit reduced fear inhibition, including poor safety-signal learning (cues predicting absence of threat) and fear extinction [4], [7], [8], [9]. These fear learning abnormalities are unique to PTSD in that they are not consistently observed in individuals who are depressed or anxious [4], [10] (Acheson et al., 2015a; Jovanovic et al., 2010). These fear inhibition deficits are linked to disrupted cortical-hippocampal circuits which putatively result in the reduced inhibition of amygdala responses observed in these patients [11], [12].

In a cross-sectional study [4], we previously reported that poor safety-signal learning and extinction were associated specifically with PTSD symptoms, not with general anxiety or depression symptoms. While these and other findings support impaired fear inhibition as a specific marker of PTSD symptoms [4], [10], it is currently unknown if the identified phenotypes evolve following trauma or constitute risk phenotypes. Evidence is mixed regarding extinction deficits being a risk factor for PTSD symptoms. Two prospective studies suggest extinction may be a risk factor in firefighters or soldiers, although these studies were relatively small with a low number of PTSD cases [13], [14]. Conversely, a twin study observed extinction deficits only in the affected twin [15]. No studies to our knowledge have examined safety-signal learning as a risk factor for PTSD. Both fear processes could play a role in increasing PTSD risk, with deficits in safety-signal learning resulting in overgeneralization of fear associations [16]. Extinction deficits may play a role in maintaining fear responding, increasing avoidance and inhibiting normal recovery after trauma.

Explicit computational models have proven highly useful in examining the specific mechanisms by which conditioning and extinction occur. The Rescorla-Wagner (RW) model, which is among the most important and influential theoretical accounts of conditioning, posits that updates in expectations are driven by prediction errors and that a learning rate parameter determines the speed of this updating processes [17], [18]. A computational modeling approach to estimate the rate of acquisition and extinction learning may therefore complement traditional behavioral analyses by isolating a theoretically relevant variable which may underpin mechanisms of PTSD risk.

Here we examined associations between safety-signal and fear-extinction learning prior to combat deployment and development of PTSD symptoms following deployment, using both traditional behavioral analyses and a computational modeling approach to estimate the rate of acquisition and extinction learning. We hypothesized that both pre-deployment safety-signal learning and pre-deployment fear-extinction learning would be impaired in participants who went on to develop PTSD relative to those who remained healthy.

## 2 Materials and methods

### 2.1 Participants

1195 infantry Marines and Navy Corpsmen enrolled in a longitudinal study of the health effects of deployment to Afghanistan and were assessed approximately 4 weeks prior to deployment (SD=22.4 days). Participants belonged to two infantry battalions, deployed for 7 months to Afghanistan between 2012 and 2013. At the time of data collection, all Marine infantry were male; thus females did not participate. Data collection occurred on a single day, with the entire testing battery (of which only a portion is presented here) being completed over approximately 4 h. Assessment of psychiatric symptoms occurred approximately 22 weeks (SD=22.4 weeks) following return. This study was approved by the institutional review boards of the University of California San Diego, VA San Diego Research Service, and the Naval Health Research Center. Written informed consent was obtained from all participants. For overall Marine Resiliency Study design and details see Baker et al., 2012 [19].

### 2.2 Fear conditioning and extinction procedure

#### 2.2.1 Apparatus

Startle pulses (108 dB, 40 ms) were delivered using a San Diego Instruments (SDI, San Diego, CA, USA) SR-HLAB Electromyography (EMG) system. Sound levels were measured using continuous tones calibrated with a Quest Sound Level Meter on the A scale, coupled to the headphones with an artificial ear. The air puff was set at 250 psi and delivered via a plastic tube positioned 2.5 cm from the center of the throat. A 48-cm monitor directly in front of the participant presented visual conditioned stimuli via E-Prime software (Psychology Software Tools, Inc., Sharpsburg, PA, USA). Presentation of the stimuli was triggered by signals from the EMG system to control synchronization of visual, acoustic, and air-puff stimuli with EMG recording.

Eyeblink EMG responses to the acoustic pulses were recorded via Ag/Ag 3 M Red Dot electrodes placed at the corner of the *orbicularis oculi* muscles at the left eye connected to the SDI SR-HLAB EMG system and laptop computer with a reference electrode at the mastoid bone [20], [21], [22]. Before electrode placement, skin was cleaned with alcohol and exfoliated with 3 M electrode prep tape. All electrode resistances were < 10 kΩ. EMG data were recorded at a sampling rate of 1 KHz, amplified (0.5 mV electrode input was amplified to 2500 mV signal output), band-pass filtered (100–1000 Hz), rectified, and smoothed with a 5-point rolling average. Expectancy responses were recorded trial-by-trial via responses on a key pad linked to E-Prime. Self-report responses were recorded at the end of each experimental phase via the same keypad.

Eyeblink data were scored as previously described in Acheson et al., 2012 [20]. In brief, eyeblink responses were examined trial by trial at a window between 100 ms before and 200 ms after pulse onset. Only responses that peaked within 100 ms of pulse onset were scored. Trials with excessive baseline noise or artifact were removed (2.1 % of trials) and imputed based on the average value of the immediately preceding and subsequent trials of the same stimulus type.

#### 2.2.2 Fear conditioning and extinction task

The fear-potentiated startle (FPS) task consisted of Acquisition and Extinction phases. Before acquisition, the participants were instructed that one colored symbol predicted the air-puff. Each phase began with 6 startle pulses in the absence of visual stimuli to stabilize startle responding. Acquisition consisted of 8 6-sec presentations of the conditioned stimulus (CS+; either a blue or yellow circle or square, balanced across subjects) that was paired with the air-puff in 75 % contingency, 8 6-sec presentations of a non-reinforced conditioned stimulus (CS-; also either a blue or yellow circle or square) that was never paired with the air-puff, and 8 presentations of the startle stimulus in the absence of visual stimuli (noise alone or “NA” trial) which measured baseline startle. The CS+ and air-puff co-terminated on reinforced trials. Startle pulses were presented 4 sec following CS+ or CS- onset. Contingency awareness was measured using a numbered keypad. Participants responded with a “1” if they expected the air puff, “2” if they were unsure, and “3” if they did not. After acquisition, participants were assessed for CS-US contingency awareness, self-reported anxiety to each CS, and subjective aversiveness of the air-puff.

After Acquisition, participants sat quietly for 5 min before beginning the Extinction phase. Participants were told to “remember what they learned” in the previous session. The Extinction phase consisted of 16 presentations of each stimulus type (CS+, CS-, and NA). No air puffs were presented. Startle pulses were delivered and ratings of expectancy were collected in the same fashion as in the Acquisition phase. After this phase, participants again rated their level of anxiety during the cues. Participants were then disconnected from the apparatus and went on to other assessment stations (see [19] for Marine Resiliency Study assessment battery).

### 2.3 Assessment of psychiatric symptoms

#### 2.3.1 Posttraumatic stress disorder

Post-traumatic stress symptoms were assessed at pre and post-deployment using a structured diagnostic interview, the Clinician Administered PTSD Scale (CAPS-IV [23]. CAPS scores range from 0 to 136 and can be used as a measure of symptom severity. Participants were defined as positive for PTSD symptoms using the partial PTSD criteria articulated by Stein and colleagues [24]. Partial PTSD criteria were chosen due to the relative psychological health of an active duty Marine cohort resulting in a fairly low number of PTSD symptoms overall and to increase statistical power for our analyses. Criteria were the presence of at least 1 B symptom, 2 C symptoms, and 2 D symptoms, with minimum frequency ratings of 1 and minimum intensity ratings of 2 (DSM-IV). Inter-rater reliability in MRS was high for both the CAPS total score (Intraclass correlation coefficient =.99) and for PTSD diagnosis (Kappa =.714). All interviews were conducted by study personnel who were trained, certified and supervised by a licensed psychiatrist (D.G.B.).

#### 2.3.2 Anxiety

Participants assessed both pre and post-deployment using the Beck Anxiety Inventory (BAI [25]) and were defined as positive for anxiety symptoms if they scored in the Moderate to Severe range (>15). The BAI is a reliable measure of general anxiety symptoms present within the past week and discriminates between anxiety vs. depressive symptoms [26].

#### 2.3.3 Depression

Participants assessed both pre and post-deployment using the Beck Depression Inventory 2 (BDI-2) [27] and were defined as positive for depression symptoms if they scored in the Moderate to Severe range (>19). The BDI-2 measures the presence of depressive symptoms within the past 2 weeks.

### 2.4 Assessment of trauma history

#### 2.4.1 Childhood trauma

Traumatic experiences during childhood were assessed at pre-deployment with a modified Childhood Trauma Questionnaire (CTQ) [28], a 34-item questionnaire (25–170 range) with strong discriminant and convergent validity.

#### 2.4.2 Life time trauma

The Life Events Checklist (LEC) [29] was used at pre-deployment to assess previous trauma history. The LEC evaluates the participant’s experience of a wide range of traumatic events. The LEC score reported here was calculated by summing all of the items scored as “happened to me” and/or “witnessed it”.

#### 2.4.3 Deployment stress

Stressful experiences during combat and deployment were assessed at 1-week post-deployment with four of the Deployment Risk and Resilience Inventory-2 (DRRI-2) scales (Post-Battle Experiences, Combat Experience, Deployment Concerns about Life and Family Disruptions, and the Difficulty Living and Working Environments subscales), with high criterion validity and internal consistency (0.92) [30]. A composite score for deployment stress was calculated to simplify analyses and conserve statistical power that might be lost by including multiple measures of the construct. Because each scale varies somewhat in score range (15–20 items), scores for each DRRI subscale were centered (subject score – group mean score) and then averaged to produce one composite DRRI score with equal weighting across scales. Positive and negative values represent higher and lower deployment stress, respectively.

### 2.5 Experimental design and statistical analysis

#### 2.5.1 Final sample

**Fig. 1:**
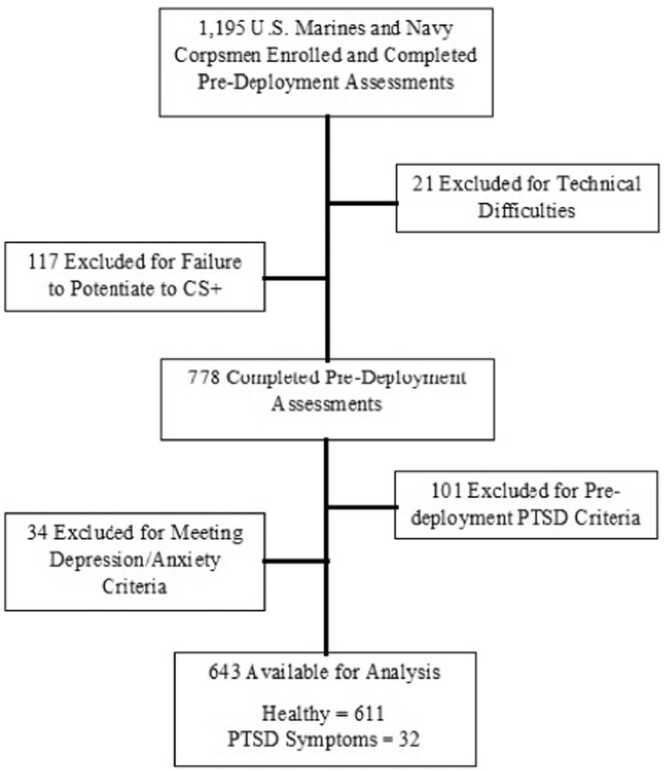
Flowchart depicting participant inclusion and exclusion across the study timeline.

Of the original 1195 Marines and Corpsmen who underwent the fear conditioning and extinction protocol, data on 21 were rendered unusable due to technical difficulties. An additional 117 (10 % of the remaining sample) were excluded from the analysis because they failed to show a CS+ response greater than baseline during the last half of the Acquisition phase. Failure to potentiate above baseline suggested that the air-puff was ineffective in inducing sufficient fear to support learning. Of the remaining 1049 participants, 778 were available for post-deployment assessment. To assess associations between pre-deployment fear conditioning and extinction performance and development of *new* symptoms following combat deployment, those participants meeting cutoffs for any symptom group at pre-deployment were additionally excluded (101 subjects). Our previous study using this cohort found that cue discrimination and extinction deficits were specific to a “pure” PTSD population without comorbid depression and generalized anxiety, thus those participants with PTSD who also met the cutoff for severe depression or generalized anxiety were excluded from the analysis presented here [4]. Estimated marginal means including “comorbid PTSD” and “Depression/Anxiety Alone” groups are included in Supplementary Materials: Tables 1–5. The remaining 643 subjects were included in the analyses, resulting in an N of 611 and 32 for Healthy and PTSD groups respectively (See Fig. 1 for exclusion flow chart). However, the N for some analyses varies due to a small number of participants having missing or invalid data for some questionnaires.

#### 2.5.2 Startle

Startle data for the Acquisition and Extinction phases were analyzed as previously described by averaging responses to each stimulus type into blocks of two trials [21], [31]. Within each block, the NA averages were subtracted from the CS+ and CS- averages to adjust for changes in baseline startle across the session. There were 4 blocks for the CS+ and CS- during the Acquisition phase, and 8 blocks for the CS+ and CS- for the Extinction phase.

To compare acquisition of CS discrimination across groups CS+ and CS- responses were averaged across the last half of the session (the first half of the session was omitted for clarity). A 2 (CS type) x 2 (group) mixed ANOVA was conducted to assess group differences. Significant interactions were followed up with alpha-adjusted post-hoc tests. To assess group differences in baseline startle, a one-way ANOVA, with appropriate post-hoc tests, was conducted on the average NA trial response across the last half of the acquisition phase.

Extinction data were analyzed by computing a measure of “% conditioned fear”, which controls for differences in fear conditioning when assessing extinction, as we have described previously for this task [4], [31]. For each subject, the maximal CS+ response during the acquisition phase is identified. A “%conditioned fear” is then calculated for each of the 8 extinction blocks using the following equation: 100*(CS+ response on extinction block/maximum response across acquisition blocks). These scores are averaged into 4-trial blocks [Early Extinction (trials 1–4), Mid Extinction 1 (trials 5–8), Mid Extinction 2 (trials 9–12), and Late Extinction (trials 13–16)]. To assess differences by symptom group, a 2 (group) x 4 (Extinction Block) a mixed ANOVA was conducted. Identical analyses were conducted on raw difference scores (included in Supplemental Materials: Table 6) to assess consistency of the results and no difference in the pattern of results was found. To assess group differences in baseline startle response during the extinction phase, a 2 (group) x 4 (Extinction Block) mixed ANOVA, with appropriate post-hoc tests, was conducted on the NA responses averaged into blocks analogous to those above.

#### 2.5.3 Computational learning models

Models were fit using Markov Chain Monte Carlo (MCMC) using the RStan interface of the Stan language [32]. We constructed four RW learning models for subsequent model comparison. The RW learning rule was used because of its relative simplicity and because of the extensive evidence for its applicability to fear conditioning [18]. The four specific models were chosen in order to examine two alterations to the basic RW model which were motivated by previous findings regarding fear conditioning. The first alteration to RW was the use of two separate learning rates (a learning rate for US+ trials and a learning rate for US- trials) during acquisition, rather than a single learning rate. This alteration was considered because separate learning rates for positive and negative prediction errors are frequently observed in the literature (e.g. [33]) and because it has specifically been observed that individuals learn more quickly from US+ than from US- trials during fear acquisition [4]. The second alteration to RW was the incorporation of a generalization learning process (in addition to a discrimination learning process) into the learning model (see below for modeling details). This alteration was motivated by previous observations that individuals do not fully discriminate between CS+ and CS- and instead partially generalize across stimuli [4]. The four models included all combinations of the presence or absence of these two alterations: 1) a model with one acquisition learning rate and discrimination-only learning, 2) a model with one acquisition learning rate and both discrimination and generalization learning, 3) a model with two acquisition learning rates and discrimination-only learning, and 4) a model with two acquisition learning rates and both discrimination and generalization learning.

In all models, the expectation of the US (air-puff) was updated on each trial according to the RW learning rule:

Expectation_subsequent_ = (1 – LR) * Expectation_current_ + LR * US,

where *LR* is a learning rate fit at the individual level and *US* is 1 for US+ trials and 0 for US- trials. In all models, startle response on each trial was modeled as depending linearly on expectation of the US, added to a baseline startle response that incorporated habituation across the task (modeled as depending linearly on trial):

Baseline = β_Intercept_ + β_Trial_ * t,

where *t* is trial, *β*_*Intercept*_ is estimated at the individual level, and *β*_*Trial*_ is estimated at the group level. For the models with discrimination-only learning, i.e. full discrimination of stimuli in the learning process, raw startle response was modeled as:

Startle ∼ normal(Baseline + β_Discrimination_ * Expectation_Discrimination_),

where *β*_*Discrimination*_ is estimated at the group level. The models with both discrimination and generalization learning were fit with two parallel learning process. In the discrimination learning process, expectations were only updated for a specific stimulus on each trial, and in the generalization learning process, expectations were updated for both CS+ and CS- on each trial. In these models, startle response was modeled as depending linearly on both discrimination expectation and generalization expectation:

Startle ∼ normal(Baseline + β_Discrimination_ * Expectation_Discrimination_ + β_Generalization_ * Expectation_Generalization_),

where *β*_*Discrimination*_ and *β*_*Generalization*_ are estimated at the group level. To select between the four constructed models, we performed model comparison using leave-one-out cross-validation [34].

#### 2.5.4 Model comparison

Model comparison favored the model with two acquisition learning rates and both discrimination and generalization learning (leave-one-out information criterion (LOOIC) = 1139589.8, standard error (SE = 811.8)) over the model with one acquisition learning rate and discrimination-only learning (LOOIC = 1143313.0, SE = 791.7), the model with one acquisition learning rate and both discrimination and generalization learning (LOOIC = 1141050.7, SE = 803.5), and the model with two acquisition learning rates and discrimination-only learning (LOOIC = 1142726.2, SE = 794.6).

#### 2.5.5 Expectancy and self-report

Expectancy responses were scored as: expect air puff = 1, unsure = 0, do not expect air puff = -1. Expectancy responses over the last half of the Acquisition phase (4 trials/stimulus type) were averaged. ANOVAs were applied to assess both task effectiveness and differences by group in the same manner as with the startle responses. Expectancy responses during the extinction phase were analyzed by 4-trial block as in the FPS analysis, with a 2 (group) x 4 (Trial Block) mixed ANOVA used to assess differences by group.

A 2 (CS type) x 2 (group) mixed ANOVA was used to assess differences in self-reported anxiety across groups. Differences across phase by group were assessed with 2 (group) x 2 (Phase) mixed ANOVA. In all analyses, significant interactions were followed up with two-tailed Tukey post-hoc tests.

We next assessed independence and relative contributions of potential predictors to symptom group membership and symptom severity. We calculated a *cue discrimination index* from the last half of the acquisition session (CS+ response – CS- response). To assess relative contributions of potential predictors, LEC, pre-deployment CAPS score, DRRI composite score, and discrimination index were entered into a multiple regression model predicting group membership with Healthy group as the reference category.

## 3 Results

### 3.1 Demographics

There were no differences across symptom groups on any of the demographic variables (Table 1).

**Table 1:** Estimated Marginal Means for Acquisition Phase Potentiated Startle Including All Symptom Groups.

	**Cue**			
	**CS+**		**CS-**	
**Post-Deployment Status**	**M**	**SE**	**M**	**SE**
Healthy	214.55	7.05	103.21	6.28
PTSD	167.66	31.00	131.05	27.58
Comorbid PTSD	258.68	46.87	58.04	41.70
Depression/Anxiety Alone	282.21	42.53	159.24	37.84

### 3.2 Trauma-history

LEC scores differed across groups [*F*(1638)= 4.97, *p* < .03] such that the PTSD group had modestly *lower* scores relative to those who remained healthy post-deployment. CTQ scores did not differ across groups [*F*(1642)= 2.47, ns]. Those who developed PTSD symptoms were more likely to report having been deployed previously relative to those who remained healthy [χ^2^(1)= 4.70, *p* < .03]. Differences between groups also emerged on deployment stress [*F*(1627)= 12.52, *p* < .001], such that the PTSD symptom group had significantly higher deployment stress during the study deployment relative to those who would remain healthy.

### 3.3 Psychiatric symptom measures

Those who would go on to develop PTSD symptoms after deployment reported elevated symptoms of PTSD and depression at the pre-deployment assessment relative to those who would remain healthy (see Table 1 for detail; omnibus tests *Fs*(1641)> 10.69, *ps*< .003). All statistical models were initially conducted with LEC, DRRI composite score, pre-deployment CAPS, and BDI-2 scores included as covariates. Since inclusion of these measures did not alter the pattern of results for any analysis, the results presented below do not include these covariates in the models. The exception of this is the logistic regression model in which we include pre-deployment CAPS score as a significant predictor in the final model.

As expected from our selection criteria, at post-deployment the PTSD group had significantly higher scores across assessment measures relative to those remaining healthy (Table 1; omnibus tests *Fs*(1641)> 22.87, *ps*< .001).

### 3.4 Comparison of task performance between psychiatric symptom groups

#### 3.4.1 Acquisition

##### 3.4.1.1 Baseline startle

There were no differences between symptom groups in average baseline startle [*F*(1, 642)< 1, ns]. This finding is consistent with previous reports indicating that baseline startle is not a risk factor for PTSD.

##### 3.4.1.2 Startle potentiation

**Fig. 2:**
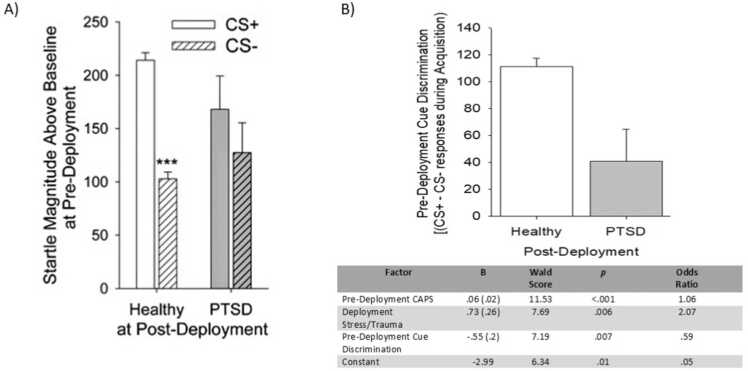
A) Pre-deployment potentiated startle across the last half of the Acquisition phase by symptom group. B) Pre-deployment differential fear score across the last half of the Acquisition phase by symptom group. Inset: table depicting odds ratios of trauma exposure and pre-deployment cue discrimination to predict PTSD symptom development at post-deployment. ***=p < .05 for CS+ vs CS- comparisons. *=p < .05 for between group comparisons. Healthy n = 611; PTSD n = 32.

The PTSD-risk group had less differential responding to the threat vs. safety cue [Fig. 2A; group x Cue *F*(1641)= 7.57, *p* < .02, partial η^2^= .012]. Post-hoc tests showed no difference between groups in response to any cue individually (*ps*>.14). This effect was further explored by calculating a cue discrimination index (CS+ response – CS- response) across participants. A one-way ANOVA confirmed that the PTSD risk group showed reduced scores relative to Healthy [Fig. 2B; *F*(1642)= 7.57, *p* < .02, partial η^2^= .012.

##### 3.4.1.3 Computational model learning rates

There were no group differences on learning rates for CS+ trials or learning rates for CS- trials [*F*s < 0.47, *ps* > .49].

##### 3.4.1.4 Expectancy and self-report

Self-reported anxiety or US expectancy during the task did not differ between groups [Table 2, Table 3]; *F*(1634)< 1, ns.

**Table 2:** Estimated Marginal Means for Extinction Phase Potentiated Startle (Percent Fear Retained) Including All Symptom Groups.

	**Extinction Block**							
	**Early**		**Mid 1**		**Mid 2**		**Late**	
**Post-Deployment Status**	**M**	**SE**	**M**	**SE**	**M**	**SE**	**M**	**SE**
Healthy	53.80	2.14	31.65	1.86	19.63	1.46	13.75	1.46
PTSD	52.92	9.35	20.26	8.16	21.33	6.41	11.59	6.38
Comorbid PTSD	52.92	13.66	48.26	11.92	23.43	9.36	28.05	9.32
Depression/Anxiety Alone	34.49	12.83	19.01	11.20	23.68	8.79	20.70	8.76

**Table 3:** Estimated Marginal Means for Acquisition and Extinction Phase Self-Reported Anxiety Including All Symptom Groups.

	**Acquisition Phase**				**Extinction Phase**			
	**Cue**				**Cue**			
	**CS+**		**CS-**		**CS+**		**CS-**	
**Post-Deployment Status**	**M**	**SE**	**M**	**SE**	**M**	**SE**	**M**	**SE**
Healthy	3.87	.09	1.19	.07	2.05	.09	-	-
PTSD	4.10	.38	1.19	.32	2.23	.41	-	-
Comorbid PTSD	4.27	.54	1.60	.46	2.13	.59	-	-
Depression/Anxiety Alone	4.71	.51	1.47	.43	2.29	.46	-	-

#### 3.4.2 Extinction

##### 3.4.2.1 Baseline startle

All groups habituated similarly to the startle stimuli across session [main effect of group [*F*(1642)< 1 < , ns; main effect of block: *F*(3,1926)= 37.67, p < .0001].

##### 3.4.2.2 Startle potentiation

**Fig. 3:**
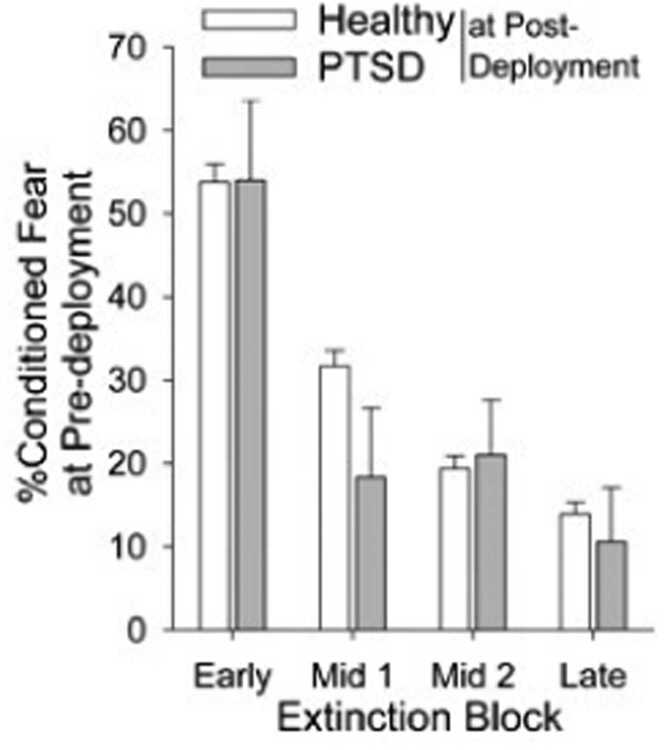
Percent conditioned fear across the Extinction phase by symptom group. Healthy n = 611; PTSD n = 32.

Both groups showed similar extinction learning [Fig. 3; Main effect of block: *F*(3,1908)= 24.98, p < .0001; group x Cue type: *F*(3,1908)< 1, ns; main effect of group: *F*(2636)< 1, ns].

##### 3.4.2.3 Computational model learning rates

Both groups showed similar extinction learning rates, *p* = .93.

##### 3.4.2.4 Expectancy and self-report

Similar to physiological extinction to the CS+ , there were no differences in US expectancy ratings across the session, with all groups reducing their expectancy by the end of extinction training [Table 3; main effect of block: F(3,1893)= 106.31, p < .0001; group X block F(3, 1893)< 1, ns]. Self-report of anxiety in response to CS+ also diminished in all subjects from acquisition to extinction phases [group x phase interaction: Table 2; *F*(1627)< 1, ns; main effect of group: *F*(2627)= 1.31, ns; main effect of testing phase: *F*(1,67)= 70.09, p < .001].

### 3.5 Independence and relative contributions of group membership predictors

To examine potential relationships between impaired differential fear conditioning and other consistent predictors of PTSD risk, exploratory correlations were calculated between a *cue discrimination index* (CS+ response – CS- response standardized), the percentage of extinction learning at Late Extinction, and scores on measures of lifetime trauma (LEC), childhood trauma (CTQ), and deployment stress (composite DRRI score). Neither cue discrimination nor late extinction index were related to any predictor measure (*rs*<.03, ns). An independent samples *t*-test was also conducted comparing those participants who had been previously deployed to those that had not. There was no significant difference between groups on cue discrimination (ts<.35, ns).

To examine the relative contributions of these potential predictors to group membership, a logistic regression was calculated with Healthy group as the reference and LEC, pre-deployment CAPS score, DRRI composite score, and discrimination index entered as predictors. LEC did not contribute significantly to the model and was thus excluded. A subsequent model was then evaluated including the interaction between cue discrimination and deployment stress, though this term also did not contribute significantly (p > .19). A final model was retained in which both pre-deployment CAPS score (β=0.06, *p* < .001, OR = 1.06), deployment stress (β=0.73, *p* < .006, OR = 2.07) and cue discrimination index (β=--0.55, *p* < .02, OR = 0.59) were predictors of PTSD group membership relative to Healthy (see Fig. 2B). A separate linear regression showed that neither Discrimination Index nor Late Extinction were significant predictors of PTSD severity (ps>.15).

**Fig. 4:**
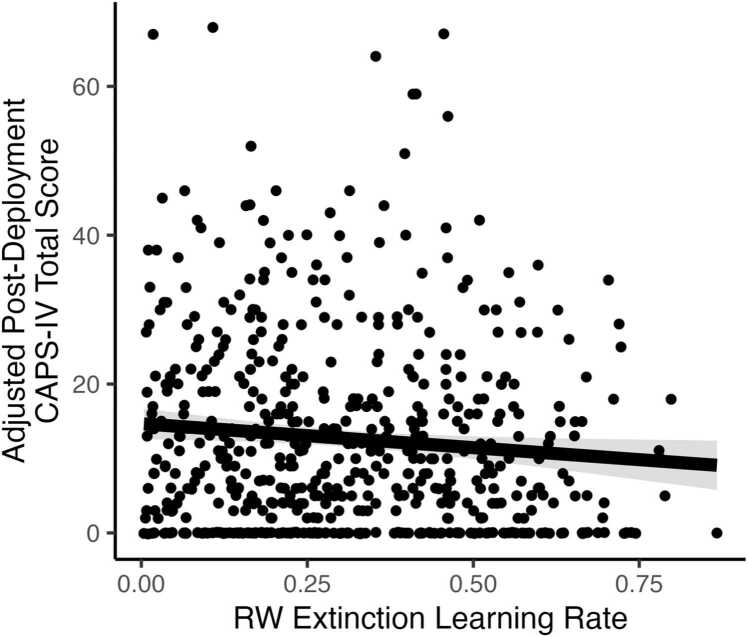
Relationship between RW extinction learning rate and post-deployment PTSD symptom severity. The plot shows the unique association between the RW extinction learning rate and post-deployment CAPS-IV total scores, adjusting for acquisition learning rates (RW Acquisition CS+, RW Acquisition CS-) in a general linear model. The solid black line represents the adjusted regression estimate for RW learning rate, with the shaded area indicating the 95 % confidence interval. Points represent partial residuals, reflecting individual variability after accounting for the effects of the other predictors. RW=Rescorla Wagner.

We further examined the predictive contribution of the RW learning rate parameters on post-deployment PTSD. First, a multivariate general linear model with the learning rate parameter for CS+ trials, learning rate parameter for CS- trials from acquisition, and the learning rate parameter for fear extinction learning was computed to predict post-deployment CAPS total symptom severity. As shown in Fig. 4, we found that the extinction learning rate parameter was negatively associated with post-deployment CAPS severity (B= −6.44 (2.86), *t* = -2.25, *p* = .025, partial η^2^= .010; see Table 4). The learning rate parameters for acquisition were not significant (Bs > -0.157, *t*s > -0.07, *ps* >.95). This pattern remained significant when controlling for pre-deployment CAPS, BDI-II, and LEC (see Supplemental Materials: Table 7). Second, we computed a logistic regression model to predict PTSD status at post-deployment using the three RW learning rate parameters. However, none of the learning rate parameters predicted PTSD status at post-deployment (*p*s > .67). These findings indicate the efficiency of extinction learning may not be specific to PTSD diagnosis, but rather predict overall trauma-related symptom severity, even after accounting for pre-deployment psychiatric and trauma history.

**Table 4:** Estimated Marginal Means for Acquisition and Extinction Phase Expectancy Ratings Including All Symptom Groups.

**Phase and Trial Block**						
**Group**	**Cue**	**Acquisition**	**Early Ext**	**Mid Ext 1**	**Mid Ext 2**	**Late Ext**
Healthy	CS+	.61 (.02)	.19 (.02)	−.32 (.03)	−.49 (.03)	−.56 (.03)
	CS-	−.78 (.02)	-	-	-	-
PTSD	CS+	.64 (.07)	.19 (.11)	−.23 (.13)	−.57 (.13)	−.59 (.12)
	CS-	−.72 (.08)	-	-	-	-
Comorbid PTSD	CS+	.60 (.10)	.28 (.15)	−.28 (.19)	−.43 (.18)	−.58 (.18)
	CS-	−.88 (.11)	-	-	-	-
Depression/Anxiety Alone	CS+	.62 (.10)	.04 (.14)	−.50 (.18)	−.66 (.17)	−.68 (.17)
	CS-	−.96 (.10)	-	-	-	-

## 4 Discussion

This is the first prospective study to examine fear conditioning, safety-signal learning and fear extinction as predictive factors for PTSD risk. As demonstrated previously [4], the FPS task was effective in producing conditioned fear and extinction learning in > 90 % of participants. Participants who remained healthy after deployment showed robust discrimination between the threat (CS+) and safety (CS-) before deployment as measured by FPS, self-reported anxiety ratings, and continuous expectancy ratings. In contrast, those participants who went on to develop PTSD showed similar levels of fear response to the safety signal as the threat signal as measured by FPS, suggesting an impairment in cue discrimination or “safety signal” learning. This impairment was also present despite intact discrimination in self-reported anxiety and expectancy ratings. This “disconnect” between the subjective perception of and automatic physiological response to fear cues is also observed in subjects with current PTSD symptoms [4], [7], [8], [9], [10]. Degree of cue discrimination also predicted PTSD group membership, though not symptom severity, with greater discrimination predicting lower risk for PTSD (OR=0.62). Conversely, extinction learning had no association with PTSD risk but the extinction learning rate, instead of magnitude, was associated with post-deployment PTSD symptom severity regardless of diagnostic status.

The pattern of reduced fear/safety discrimination in participants at risk for PTSD symptoms is similar to that demonstrated in those with current PTSD symptoms [4], [8], [10]. This pattern suggests that reduced discrimination learning, as measured by FPS, may represent a stable risk factor that is present both prior to emergence of, and during the expression of PTSD symptoms. Of note, reduced fear/safety discrimination was not related to other known risk factors (lifetime trauma, childhood trauma, and deployment stress), suggesting this measure probes an independent mechanism. This pattern of results further suggests that reduced fear/safety discrimination was not a consequence of prior traumas, and may be driven by genomic or unidentified environmental influences. Impaired fear-safety discrimination has also been reported in panic disorder [35] and social anxiety disorder [36], though not in anxiety disorders as a whole [37]. The specificity of cue discrimination deficits in only certain anxiety disorders raises the possibility that the impaired cue discrimination learning may represent a risk marker for a “fear-based” subset of anxiety disorders.

Consistent with our hypothesis, slower pre-deployment fear extinction learning predicted post-deployment PTSD symptoms. Current PTSD symptoms are consistently associated with poor extinction learning in the FPS task [4], [9], and decreased pre-trauma fear extinction learning has been shown to predict the development of PTSD in previous studies [13], [14]. In contrast, a previous twin study reporting that reduced recall of fear extinction memory (and associated hypo-activation of prefrontal cortex) is an acquired feature of PTSD [15], not a pre-existing risk factor. Importantly, unlike our model-based analysis, our model-free analysis of fear extinction in which trials were grouped into four blocks did not find a significant difference between individuals who developed post-deployment PTSD and individuals who did not. This suggests that a computational analysis examining a specific, theoretically motivated construct (rate of extinction learning) may have significant utility in better capturing mechanisms underlying risk for PTSD. [38]. A slower extinction rate may reflect impairments in predictive updating or reinforcement learning, which have been linked to elevated stress and anxiety [39]. These learning deficits may occur even when fear magnitude appears normal, suggesting that trial-by-trial modeling provides a more sensitive marker of maladaptive extinction learning than static fear responses alone [40].

The current findings have implications for understanding the neurobiology of PTSD risk. While the neural substrates for cue discrimination/safety signal learning are not well known, potential circuits have been identified which include the amygdala, ventromedial prefrontal cortex, and substantial contributions from the hippocampus [41], [42]. Insight into the neural circuitry involved in the type of learning examined here may also be gained from studies on conditioned fear cue generalization/discrimination, or the extent to which a fear response is maintained when an organism is presented with stimuli that are more-or-less similar to the original threat cue. Animal research using hippocampal lesions and human research using fMRI imaging suggests the pattern separation function of the hippocampus is central to distinguishing between similar cues representing threat vs. safety [43], [44], [45]. The suggestion of contributions from the hippocampus is intriguing given evidence that hippocampal volume and function may play a role in risk for development of PTSD following trauma exposure [12], [46], [47], [48]. Lissek and colleagues [49] examined patterns of fMRI activation in response to gradually differing visual threat cues in humans and identified a broader number of regions involved including the dorsal and ventral prefrontal cortex, as well as the ventral hippocampus, involved in cue generalization/discrimination learning. Taken as a whole, these findings suggest a potential model of PTSD vulnerability which involves impaired pattern separation in the hippocampus resulting a lack of PFC-mediated fear inhibition in the presence of cues that are similar yet distinct from those predicting threat. However, this model runs contrary to other recent models of PTSD vulnerability which suggest hippocampal dysfunction is an acquired feature [11]. The issue of the pre-existing vs. acquired nature of hippocampal dysfunction in PTSD and its consequences will continue be an important topic for future research, as it may be an important target for preventative and treatment efforts.

Limitations of the current study must be noted. First, we utilized a highly screened sample of male Marines and Navy corpsmen, which may limit generalizability [50] and resulted in a fairly low occurrence of PTSD symptoms even given our subthreshold criteria. Future research is needed to determine if safety signal learning and extinction learning rate are potential predictors in women or in PTSD subjects with different trauma types. There was also a significant difference in the proportion of participants in each group who had experienced a prior deployment. However, having had a prior deployment did not alter safety-signal learning performance relative to those who had not been deployed, and including prior deployment in the analyses did not alter the pattern of results. The PTSD symptom group also differed from the healthy group in intensity of combat experience. While combat intensity could not have retroactively influenced task performance, combat intensity and safety-signal learning performance nevertheless did not correlate, suggesting that cue discrimination was not associated with risk for future trauma exposure.

In conclusion, this was a large prospective study of fear learning processes that may contribute to PTSD risk. Discrimination of fear vs. safety cues as well as rate of fear extinction learning before trauma exposure were significantly associated with PTSD symptom development after trauma. These data support further examination of safety signal learning, cue discrimination, and extinction learning rate as potential intermediate phenotypes, alongside other potential psychophysiological risk markers [51], to identify mechanisms underlying PTSD risk and resiliency. Further, the paradigm used in the current study may represent a candidate paradigm for screening potential prophylactic or therapeutics aimed at safety-learning and extinction learning deficits.

## Significance statement

This prospective study of PTSD risk factors demonstrates that cue discrimination/generalization as well as extinction learning deficits predict development of PTSD after trauma exposure. This is the first study to indicate that both cue discrimination and rate of fear extinction may be cognitive traits by which some individuals develop chronic PTSD after fear exposure. Cue discrimination/overgeneralization and rate of extinction learning therefore may play a mechanistic role in trauma processing and offer potential phenotypes for further mechanistic work to understand the circuits and substrates underlying PTSD risk.

## Declaration of Competing Interest

The authors declare the following financial interests/personal relationships which may be considered as potential competing interests: Dean Acheson, Dewleen Baker, Caroline Nievergelt, Victoria Risbrough, Mark Geyer reports financial support was provided by Veterans Integrated Services Network 22 Center of Excellence for Stress and Mental Health. Victoria Risbrough reports financial support was provided by US Department of Veterans Affairs [BX006186, CX002343], 10.13039/100000065National Institute of Neurological Disorders and Stroke (NINDS) [NS135620] and 10.13039/100000005Department of Defense (DOD) [HT94252510832]. Stout [CX002760], Howlett [CX002872] and Harle [CX002456] reports financial support was provided by US Department of Veterans Affairs. Mark Geyer reports a relationship with San Diego Instruments Inc that includes: equity or stocks. If there are other authors, they declare that they have no known competing financial interests or personal relationships that could have appeared to influence the work reported in this paper.

The contents do not represent the views of the U.S. Department of Veteran Affairs or the United States Government.

## Footnotes

Appendix A Supplementary data associated with this article can be found in the online version at doi:10.1016/j.xjmad.2025.100148.
