# Supplementary material for: A prospective study of pre-trauma fear learning and extinction as risk factors for posttraumatic stress disorder
Source: J Mood Anxiety Disord. 2025 Sep 13;12:100148. doi: 10.1016/j.xjmad.2025.100148 (PMC12524147; doi:10.1016/j.xjmad.2025.100148)
Supplement: Supplementary file 1 — Supplementary material [file mmc1.docx]

**Table 1. Estimated Marginal Means for Acquisition Phase Potentiated Startle Including All Symptom Groups**

|  | **Cue** | | | |
| --- | --- | --- | --- | --- |
|  | **CS+** | | **CS-** | |
| **Post-Deployment Status** | **M** | **SE** | **M** | **SE** |
| Healthy | 214.55 | 7.05 | 103.21 | 6.28 |
| PTSD | 167.66 | 31.00 | 131.05 | 27.58 |
| Comorbid PTSD | 258.68 | 46.87 | 58.04 | 41.70 |
| Depression/Anxiety Alone | 282.21 | 42.53 | 159.24 | 37.84 |

**Table 2. Estimated Marginal Means for Extinction Phase Potentiated Startle (Percent Fear Retained) Including All Symptom Groups**

|  | **Extinction Block** | | | | | | | |
| --- | --- | --- | --- | --- | --- | --- | --- | --- |
|  | **Early** | | **Mid 1** | | **Mid 2** | | **Late** | |
| **Post-Deployment Status** | **M** | **SE** | **M** | **SE** | **M** | **SE** | **M** | **SE** |
| Healthy | 53.80 | 2.14 | 31.65 | 1.86 | 19.63 | 1.46 | 13.75 | 1.46 |
| PTSD | 52.92 | 9.35 | 20.26 | 8.16 | 21.33 | 6.41 | 11.59 | 6.38 |
| Comorbid PTSD | 52.92 | 13.66 | 48.26 | 11.92 | 23.43 | 9.36 | 28.05 | 9.32 |
| Depression/Anxiety Alone | 34.49 | 12.83 | 19.01 | 11.20 | 23.68 | 8.79 | 20.70 | 8.76 |

**Table 3. Estimated Marginal Means for Acquisition and Extinction Phase Self-Reported Anxiety Including All Symptom Groups**

|  | **Acquisition Phase** | | | | **Extinction Phase** | | | |
| --- | --- | --- | --- | --- | --- | --- | --- | --- |
|  | **Cue** | | | | **Cue** | | | |
|  | **CS+** | | **CS-** | | **CS+** | | **CS-** | |
| **Post-Deployment Status** | **M** | **SE** | **M** | **SE** | **M** | **SE** | **M** | **SE** |
| Healthy | 3.87 | .09 | 1.19 | .07 | 2.05 | .09 | - | - |
| PTSD | 4.10 | .38 | 1.19 | .32 | 2.23 | .41 | - | - |
| Comorbid PTSD | 4.27 | .54 | 1.60 | .46 | 2.13 | .59 | - | - |
| Depression/Anxiety Alone | 4.71 | .51 | 1.47 | .43 | 2.29 | .46 | - | - |

**Table 4. Estimated Marginal Means for Acquisition and Extinction Phase Expectancy Ratings Including All Symptom Groups**

| **Phase and Trial Block** | | | | | | |
| --- | --- | --- | --- | --- | --- | --- |
| **Group** | **Cue** | **Acquisition** | **Early Ext** | **Mid Ext 1** | **Mid Ext 2** | **Late Ext** |
| Healthy | CS+ | .61 (.02) | .19 (.02) | -.32 (.03) | -.49 (.03) | -.56 (.03) |
|  | CS- | -.78 (.02) | - | - | - | - |
| PTSD | CS+ | .64 (.07) | .19 (.11) | -.23 (.13) | -.57 (.13) | -.59 (.12) |
|  | CS- | -.72 (.08) | - | - | - | - |
| Comorbid PTSD | CS+ | .60 (.10) | .28 (.15) | -.28 (.19) | -.43 (.18) | -.58 (.18) |
|  | CS- | -.88 (.11) | - | - | - | - |
| Depression/Anxiety Alone | CS+ | .62 (.10) | .04 (.14) | -.50 (.18) | -.66 (.17) | -.68 (.17) |
|  | CS- | -.96 (.10) | - | - | - | - |

**Table 5. Estimated Marginal Means for Rescorla Wagner Model Learning Rate Parameters Including All Symptom Groups**

| **Rescorla Wagner Model Learning Rate Parameters** | | | |
| --- | --- | --- | --- |
| **Group** | **RW LR CS+** | **RW LR CS+** | **RW Extinction LR** |
| Healthy | .45 (.01) | .45 (.01) | .31 (.01) |
| PTSD | .41 (.06) | .48 (.04) | .29 (.04) |
| Comorbid PTSD | .55 (.09) | .51(.06) | .26 (.06) |
| Depression/Anxiety Alone | .54 (.09) | .32 (.06) | .38 (.05) |

**Table 6. Extinction Phase Analysis on Raw Difference Scores**

Both groups showed similar extinction learning when analyzing raw potentiated startle values [Table X; Main effect of block: *F*(3,1908)=33.81, p<.001; group x Cue type : *F*(3,1908)<1, ns; main effect of group: *F*(2,636)=1.06, ns].

|  | **Extinction Block** | | | | | | | |
| --- | --- | --- | --- | --- | --- | --- | --- | --- |
|  | **Early** | | **Mid 1** | | **Mid 2** | | **Late** | |
| **Post-Deployment Status** | **M** | **SE** | **M** | **SE** | **M** | **SE** | **M** | **SE** |
| Healthy | 193.15 | 7.35 | 127.03 | 6.84 | 75.74 | 5.32 | 58.98 | 5.00 |
| PTSD | 176.58 | 32.07 | 90.12 | 29.85 | 77.63 | 23.24 | 24.11 | 21.85 |

| **Table 7. General linear model of RW learning rate parameters predicting post-deployment CAPS symptom severity after controlling for pre-deployment CAPS, BDI-2, and LEC.** | | | |
| --- | --- | --- | --- |
| Variable | B | *p* | partial η^2^ |
| Intercept | 12.56 (.50) | <.001 | 0.157 |
| CAPS total score (pre) | 0.398 (.06) | <.001 | 0.065 |
| BDI-2 total score (pre) | 0.395 (.14) | .005 | 0.014 |
| LEC total score (pre) | -0.108 (.05) | .031 | 0.008 |
| RW LR CS+ | 1.354 (3.1) | .663 | 0.000 |
| RW LR CS- | 0.812 (2.2) | .705 | 0.000 |
| RW Extinction LR | -6.34 (2.6) | .016 | 0.010 |
| Standard errors in parentheses. RW = Rescorla Wagner computational model; CAPS=Clinician Administered PTSD Scale; pre = pre-deployment.; BDI-2=Beck Depression Inventory-2; LEC=lifetime Events Checklist; LR=learning rate parameter. | | | |
